# A new rain frog of the genus *Pristimantis* (Anura, Craugastoridae) from the Cordillera del Cóndor, southeastern Ecuador

**DOI:** 10.3897/zookeys.1282.187506

**Published:** 2026-06-15

**Authors:** Elías Figueroa-Coronel, Diego F. Cisneros-Heredia, David Brito-Zapata, Julio C. Carrión-Olmedo, Carolina Reyes-Puig

**Affiliations:** 1 Universidad San Francisco de Quito USFQ, Colegio de Ciencias Biológicas y Ambientales, Instituto de Biodiversidad Tropical IBIOTROP, Laboratorio de Zoología Terrestre, Museo de Zoología, Quito 170901, Ecuador Environmental Futures Research Centre, School of Science, University of Wollongong Wollongong Australia; 2 Instituto Nacional de Biodiversidad, Unidad de Investigación, Quito, Ecuador Universidad San Francisco de Quito USFQ, Colegio de Ciencias Biológicas y Ambientales, Instituto de Biodiversidad Tropical IBIOTROP, Laboratorio de Zoología Terrestre, Museo de Zoología Quito Ecuador; 3 Environmental Futures Research Centre, School of Science, University of Wollongong, Wollongong, NSW 2500, Australia Instituto Nacional de Biodiversidad, Unidad de Investigación Quito Ecuador; 4 Smithsonian’s National Zoo and Conservation Biology Institute, Center for Species Survival, Front Royal, Virginia 22630, USA Smithsonian’s National Zoo and Conservation Biology Institute, Center for Species Survival Front Royal United States of America; 5 Fundación EcoMinga, Red de Protección de Bosques Amenazados, Quito, Ecuador Fundación EcoMinga, Red de Protección de Bosques Amenazados Quito Ecuador

**Keywords:** Amphibia, forearm tubercles, *
Huicundomantis
*, sub-Andean cordilleras

## Abstract

A new species of *Pristimantis* is described from the Cordillera del Cóndor, Zamora Chinchipe Province, southeastern Ecuador, based on morphological and molecular data, *Pristimantis etsa***sp. nov**. The new species is diagnosed from its congeners by the following combination of characters: female SVL 32.5 mm and male SVL 18.3 mm, dorsolateral folds formed by rows of subconical tubercles, strongly areolate ventral skin, two distinct rows of forearm tubercles, one along the ventrolateral margin and a second along the externolateral margin of the forearm, and a prominent yellow groin blotch in the female holotype. The species belongs to the *Pristimantis cryptomelas* group, part of the *Huicundomantis* subgenus, and is closely related to *P. nangaritza*, *P. verrucosus*, and *P. plateado*. Currently, the species is known only from its type locality, where it inhabits low montane evergreen forests at elevations of 1,655–1,830 m. Additionally, we discuss the use of the term “ulnar tubercles” in *Pristimantis*, noting that it may refer to tubercles occupying different positions on the forearm.

## Introduction

*Pristimantis* Jiménez de la Espada, 1870 is a hyperdiverse genus of Brachycephaloidea Günther, 1858 within the family Craugastoridae Hedges, Duellman & Heinicke, 2008 and subfamily Strabomantinae Hedges, Duellman & Heinicke, 2008 (Portik et al. 2023), currently comprising 635 described species (Frost 2026). The genus is broadly distributed across Central and South America, where it occupies a wide range of humid tropical, subtropical, and montane ecosystems (Heinicke et al. 2007; Pinto-Sánchez et al. 2012). Ecuador represents one of the main centers of diversity for the genus, harboring 274 species of *Pristimantis*, 174 of which are endemic (Frost 2026). Within Ecuador, *Pristimantis* occurs across a broad environmental gradient, from the Amazonian and Pacific lowlands to the páramo of the high Andes. Their distribution also includes the sub-Andean cordilleras of Guacamayos, Kutukú, and Cóndor, east of the Andes of Ecuador, where diversity remains comparatively understudied (Terán-Valdez and Guayasamin 2010; Ortega-Andrade et al. 2015; Yánez-Muñoz et al. 2019; Brito-Zapata et al. 2021, 2024).

The Cordillera del Cóndor, located in southeastern Ecuador and northern Peru, is the largest and oldest of Ecuador’s sub-Andean ranges. It was shaped by Mesozoic and early Cenozoic uplifts preceding the final orogenic folding of the northern Andes, followed by extensive summit erosion that produced characteristic tabletop massifs (Terán 1989; Neill 2005). The complex geological history has resulted in strong altitudinal zonation and exceptional conditions for high endemism across multiple taxa, including anurans, with numerous species described from the mountain range in recent years (Neill 2005; Ron et al. 2018; Brito-Zapata and Reyes-Puig 2021; Brito-Zapata et al. 2021; Molino et al. 2025). Here, we describe a new species of *Pristimantis* discovered during a 2023 expedition to the Río Blanco region on the western slopes of the Cordillera del Cóndor in Ecuador, led by the Laboratorio de Zoología Terrestre of the Universidad San Francisco de Quito.

## Materials and methods

### Classification and species concept

We follow Heinicke et al. (2018), Barrientos et al. (2021), and Motta et al. (2021) for family-level classification within Brachycephaloidea. We adopt the General Lineage Concept of species, considering species as independently evolving lineages within a metapopulation supported by operational criteria (de Queiroz 2005, 2007).

### Comparative specimens

We obtained morphological data from specimens deposited at the following collections: División de Herpetología, Instituto Nacional de Biodiversidad, Quito (**DHMECN**); División de Anfibios, Museo de Zoología de la Pontificia Universidad Católica del Ecuador (**QCAZ**), Museo de Zoología, Universidad San Francisco de Quito, Quito (**ZSFQ**). Information on species for comparative diagnoses was obtained from examined specimens (see Appendix 1) and literature, including original species descriptions.

### Fieldwork

Fieldwork was conducted in August 2023 near the Río Blanco hamlet (3°54'17.28"S, 78°30'27.36"W, 1560–2350 m elevation), a remote location accessible by car on the western slope of the Cordillera del Cóndor, Zamora Chinchipe Province, Ecuador. Live individuals were photographed, euthanized with 2% lidocaine, fixed in 8% formalin, and preserved in 75% ethanol. Tissues were preserved in 95% ethanol at −20 °C. Localities, elevations, and coordinates were recorded on field datasheets and with a Garmin handheld GPS. Voucher specimens are deposited in the Museo de Zoología, Universidad San Francisco de Quito (**ZSFQ**).

### Morphological analysis

For terminology and measurements, we followed Lynch and Duellman (1997) and Duellman and Lehr (2009). We recorded the following measurements with a digital caliper (resolution 0.01 mm), rounded to the nearest 0.1 mm: snout-vent length (**SVL**), tibia length (**TL**), head width (**HW**), head length (**HL**), interorbital distance (**IOD**), width of upper eyelid (**EW**), internarial distance (**IND**), eye-nostril distance (**EN**), tympanum diameter (**TD**), eye diameter (**ED**), hand length (**HaL**) and foot length (**FL**). Color in life was described from field notes and *in situ* photographs. Sex was determined by secondary sexual characteristics (i.e., nuptial pads) and direct gonadal inspection. The scientific illustrations were prepared from photographs of the holotype, both in life and in preservative, supplemented by detailed explanations of the observed morphological features.

### Phylogenetic analysis

We generated two overlapping sequences from a single individual (male paratype ZSFQ 6189) flanking the partial mitogenome from 12S rRNA to ND1. First, we targeted an ~2000 bp fragment from 12S rRNA up to a partial fragment of 16S rRNA using 12sL4E (TACACATGCAAGTYTCCGC) and 16H36E (AAGCTCCAWAGGGTCTTCTCGTC) (Heinicke et al. 2007), with the following thermocycler protocol: 5 min at 95 °C, then 35 cycles of: 45s at 95 °C, 35s at 50 °C, and 2 min at 72 °C, then 5 min at 72 °C. Then, we amplified an overlapping fragment of ~2500 bp long from 16S rRNA up to NADH dehydrogenase subunit 1 gene (ND1) using 16L19 (AATACCTAACGAACTTAGCGATAGCTGGTT) and t-Met-frog (TTGGGGTATGGGCCCAAAAGCT) (Wiens et al. 2005; Moen and Wiens 2009) with the following thermocycler protocol: initial denaturation 5 min at 95 °C, 30s at 95 °C, 30s at 57 °C and 4 min at 72 °C for 35 cycles, with a final extension time of 5 min at 72 °C. We followed the DNA extraction, PCR amplification, Nanopore sequencing, and bioinformatics methodology described by Reyes-Puig et al. (2024) and Yánez-Muñoz et al. (2025). All these procedures were performed at the Biotechnology Laboratory of the Instituto Nacional de Biodiversidad (INABIO) in Quito, Ecuador. Sequencing was performed on a MinION mk1c using Flongle Flow Cells R10.4.1 and the Rapid Barcoding Kit 96 V14 (SQK-RBK114.96) following the manufacturer’s protocols. Raw reads were high-accuracy (HAC) basecalled and demultiplexed with Dorado 7.3.11. Consensus sequences were generated with NGSpeciesID (Sahlin et al. 2021) using the above Q12 fastq reads. The sequence generated in this study was deposited in GenBank (accession number: PZ403757). To place our newly sequenced sample within a broader phylogenetic context, we first conducted a BLASTn search on NCBI GenBank using our raw sequences as queries. The top matching sequences (highest identity and query coverage) were retrieved and aligned with our dataset using MUSCLE (Edgar 2004). We reconstructed an exploratory phylogenetic tree in IQ-TREE v. 2 (Minh et al. 2020) to determine the general placement of our samples. Upon confirming that our sequences nested robustly within the subgenus *Huicundomantis* Páez & Ron, 2019, we subsequently refined our taxon sampling. We restricted our final dataset to include all available representative sequences of *Huicundomantis* (*sensu*Páez and Ron 2019; Székely et al. 2020, 2026b). We included the species within the *P. cryptomelas* group Páez & Ron, 2019: *P. gagliardoi* Bustamante & Mendelson, 2008, *P. muscosus* (Duellman & Pramuk, 1999), *P. spinosus* (Lynch, 1979), *P. cryptomelas* (Lynch, 1979), *P. nunezcortezi* Chávez et al., 2025, *P. nangaritza* Páez & Ron, 2019, *P. verrucosus* Székely et al., 2026a, and *P. plateado* Székely et al., 2026. We also included the species within the *P. phoxocephalus* clade: *P. torresi* Páez & Ron, 2019, *P. lojanus* Székely et al., 2021, *P. phoxocephalus* (Lynch, 1979), *P. totoroi* Páez & Ron, 2019, *P. chinguelas* Chávez et al., 2025, *P. multicolor* Páez & Ron, 2019, *P. percultus* (Lynch, 1979), *P. chomskyi* Páez & Ron, 2019, *P. andinogigas* Yánez-Muñoz et al., 2019, *P. melanops* Székely et al., 2026, *P. oculolineatus* Székely et al., 2026, *P. lutzae* Páez & Ron, 2019, *P. gloria* Páez & Ron, 2019, *P. jimenezi* Páez & Ron, 2019, *P. balionotus* (Lynch, 1979), *P. hampatusami* Yánez-Muñoz et al., 2016, *P. verrucolatus* Páez & Ron, 2019, *P. tinguichaca* Brito et al., 2016, *P. versicolor* (Lynch, 1979), *P. atillo* Páez & Ron, 2019, *P. teslai* Páez & Ron, 2019, *P. atratus* (Lynch, 1979), *P. chusquea* Székely et al., 2026, and *P. translucidus* Székely et al., 2026. We used species from the *Pristimantis lacrimosus* group and *Pristimantis orestes* group as outgroups. We used the updated matrix for the subgenus *Huicundomantis* available from Székely et al. (2026b) and aligned our 12S rRNA and 16S rRNA fragments. The matrix was visually inspected in Mesquite 3.81 (Maddison and Maddison 2018) and aligned with MUSCLE (Edgar 2004). Phylogenetic analyses were conducted using a multiple sequence alignment in PHYLIP format. Maximum Likelihood inference was performed with IQ-TREE v2 (Minh et al. 2020). The best-fitting nucleotide substitution model was specified as the General Time-Reversible model with optimized state frequencies (-m GTR+FO) option based on the model reported by Székely et al. (2026b). Branch support was assessed using 10,000 replicates of ultrafast bootstrap approximation (UFBoot; Hoang et al. 2018) (-bb 10000) and 5,000 replicates of the SH-like approximate likelihood ratio test (SH-aLRT) (-alrt 5000). Individual bootstrap trees were saved using the option -wbt. To ensure a thorough search of the tree space, the perturbation strength was set to 0.5 (-pers 0.5) and the candidate clustering stop condition was set to 100 (-numstop 100). To ensure reproducibility, a fixed random seed of 255703 was applied (-seed 255703). All analyses were executed under these specified parameters, and the resulting consensus tree was used for downstream phylogenetic interpretation. Support values mentioned herein follow this format: SH-aLRT support (%)/ultrafast bootstrap support (%). Uncorrected p-distances were calculated with a 933 bp-long fragment of 16S rRNA using MEGA 11 (Tamura et al. 2021).

### Conservation risk assessment

We assessed extinction risk in accordance with the IUCN Red List Categories and Criteria (IUCN 2012) and the IUCN Standards and Petitions Committee (2024). The extent of occurrence and area of occupancy (cell size 2 km) were calculated using GeoCAT (Bachman et al. 2011).

## Results

### Molecular phylogenetic analyses

**Figure 1.**
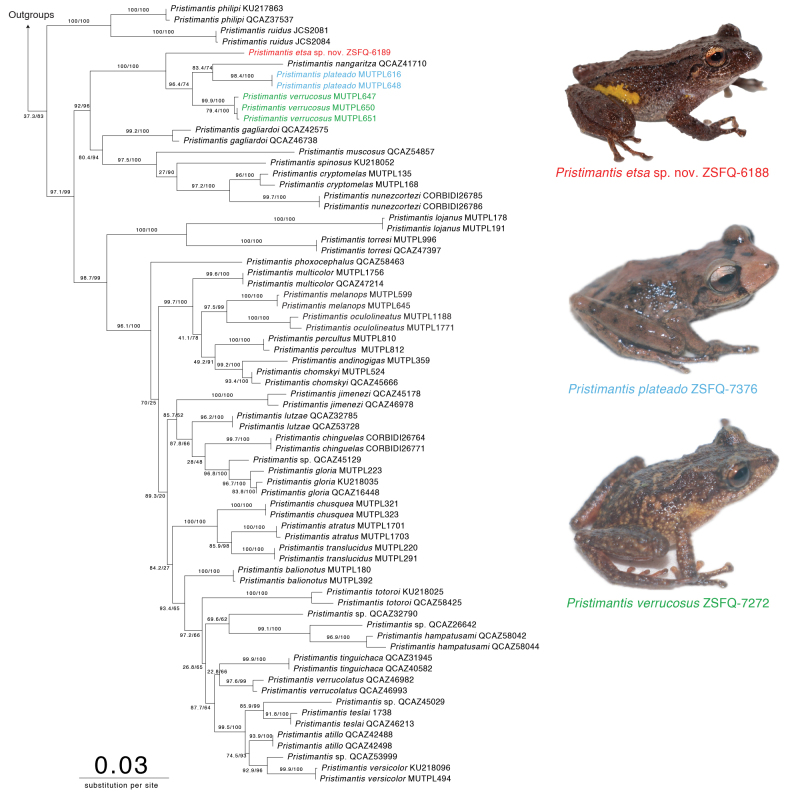
Maximum-likelihood phylogeny of *Pristimantis* inferred from concatenated mitochondrial 12S and 16S sequences. The tree shows the phylogenetic placement of *Pristimantis etsa* sp. nov. within the subgenus *Huicundomantis*. Numbers at nodes indicate SH-aLRT support (%)/ultrafast bootstrap support (%); only support values for principal nodes are shown. Outgroups are not shown. The scale bar indicates substitutions per site. Museum catalogue numbers are shown after species names. Photographs show, from top to bottom, *P. etsa* sp. nov., holotype ZSFQ 6188, *P. plateado*ZSFQ 7376, and *P. verrucosus*ZSFQ 7272. Photographs by Carolina Reyes-Puig.

**Figure 2.**
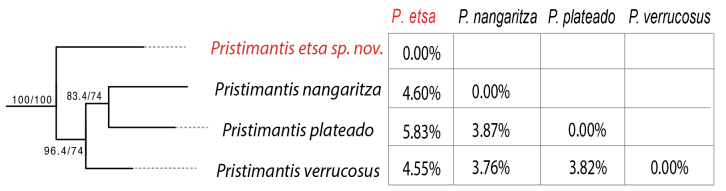
Phylogenetic placement and pairwise genetic distances of *Pristimantis etsa* sp. nov. and closely related species of the *P. cryptomelas* group. Left, simplified maximum-likelihood topology showing the relationship of *P. etsa* sp. nov. with *P. nangaritza*, *P. plateado*, and *P. verrucosus*; values at nodes indicate SH-aLRT/UFBoot support. Right, pairwise uncorrected genetic distances among the same taxa, expressed as percentages. *Pristimantis etsa* sp. nov. is highlighted in red.

The final aligned and concatenated mitochondrial DNA (mtDNA) dataset consisted of a character matrix of 92 individuals spanning a total length of 1,857 base pairs (bp). This multilocus matrix comprised a 924 bp fragment of the 12S rRNA gene and a 933 bp fragment of the 16S rRNA gene. The optimal tree search reached a best log-likelihood score of −19,593.514 (consensus tree lnL = −19,596.734). The Maximum Likelihood analysis recovered a well-resolved phylogenetic hypothesis for *Pristimantis etsa* sp. nov., placing it within the genus *Pristimantis* (Figs 1, 2). The topology obtained was consistent across bootstrap replicates and showed no major conflicts among well-supported nodes. *Pristimantis etsa* sp. nov. was recovered as sister to the clade formed by *P. nangaritza*, *P. plateado*, and *P. verrucosus*, with strong support (100/100). This clade was recovered as a sister to the clade composed of *Pristimantis gagliardoi*, *P. muscosus*, *P. spinosus*, *P. cryptomelas*, and *P. nunezcortezi* (92/96 branch support values). Uncorrected 16S p-distances between *P. etsa* sp. nov. and its closest relatives were 4.60% for *P. nangaritza*, 4.55% for *P. verrucosus*, and 5.83% for *P. plateado*.

### Taxonomic account

#### Pristimantis etsa sp. nov.

Taxon classificationAnimaliaAnuraCraugastoridae

FC79951E-B916-5D77-94F8-89410A51D2FE

https://zoobank.org/911339D0-544E-484A-8F4E-84C514291B53

**Figure 3.**
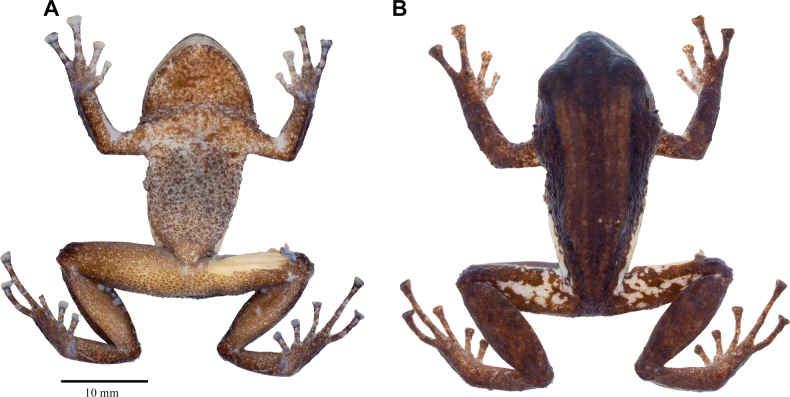
Preserved holotype of *Pristimantis etsa* sp. nov., ZSFQ 6188, adult female, SVL = 32.5 mm. **A**. Ventral view; **B**. Dorsal view.

**Figure 4.**
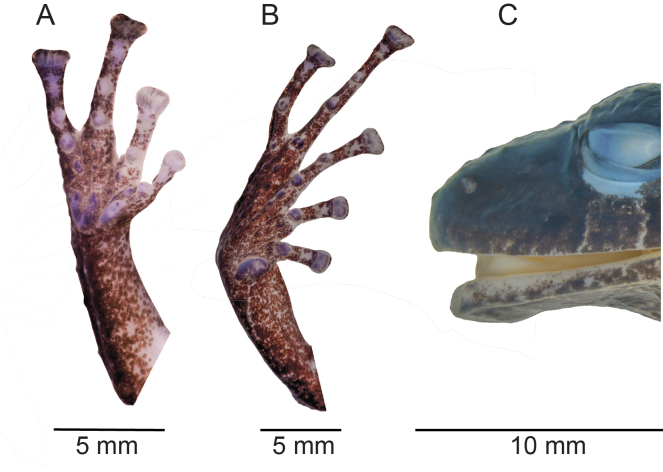
Detail of holotype (ZSFQ 6188) of *Pristimantis etsa* sp. nov., adult female, SVL = 32.5 mm. **A**. Hand; **B**. Foot; **C**. Lateral view of head.

**Figure 5.**
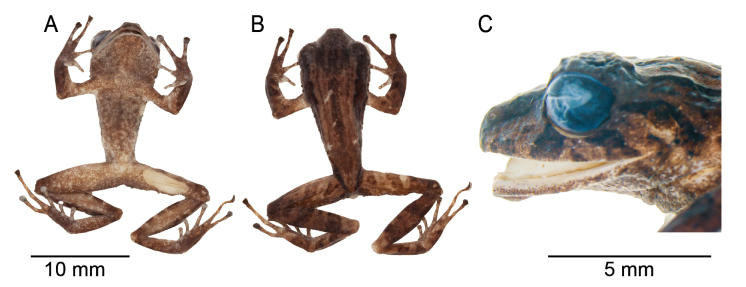
Preserved paratype of *Pristimantis etsa* sp. nov., adult male, ZSFQ 6189, SVL = 18.3 mm in (**A**) ventral view, (**B**) dorsal view, and (**C**) details of head.

**Figure 6.**
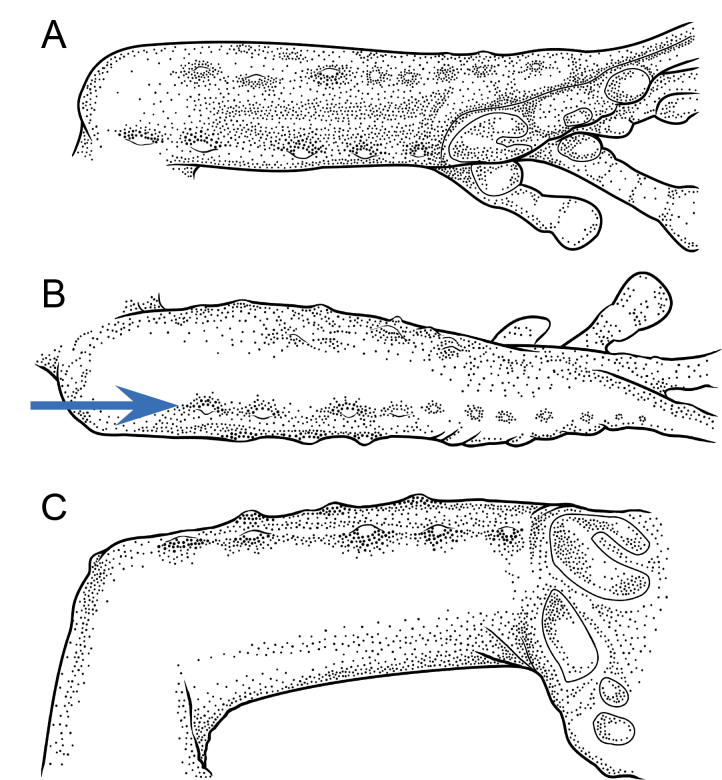
Two distinct rows of ‘ulnar’ tubercles present in *Pristimantis etsa* sp. nov. (ZSFQ 6188, adult female, holotype). In the traditional sense, the ulnar line of tubercles extends ventrolaterally from the base of the palmar tubercle to near the elbow. The second line of tubercles, indicated by the blue arrow, extends externolaterally along the postaxial margin of the forearm. Drawings by Rita Hidalgo.

**Figure 7.**
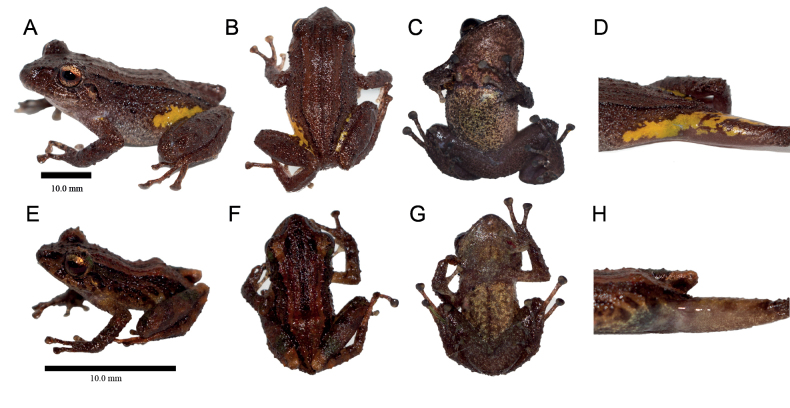
Color in life of *Pristimantis etsa* sp. nov. **A, E**. Dorsolateral view; **B, F**. Dorsal view; **C, G**. Ventral view, and **D, H**. Groin. (**A–D**) ZSFQ 6188; (**E–H**) ZSFQ 6189. Photographs by David Brito-Zapata and Carolina Reyes-Puig.

**Figure 8.**
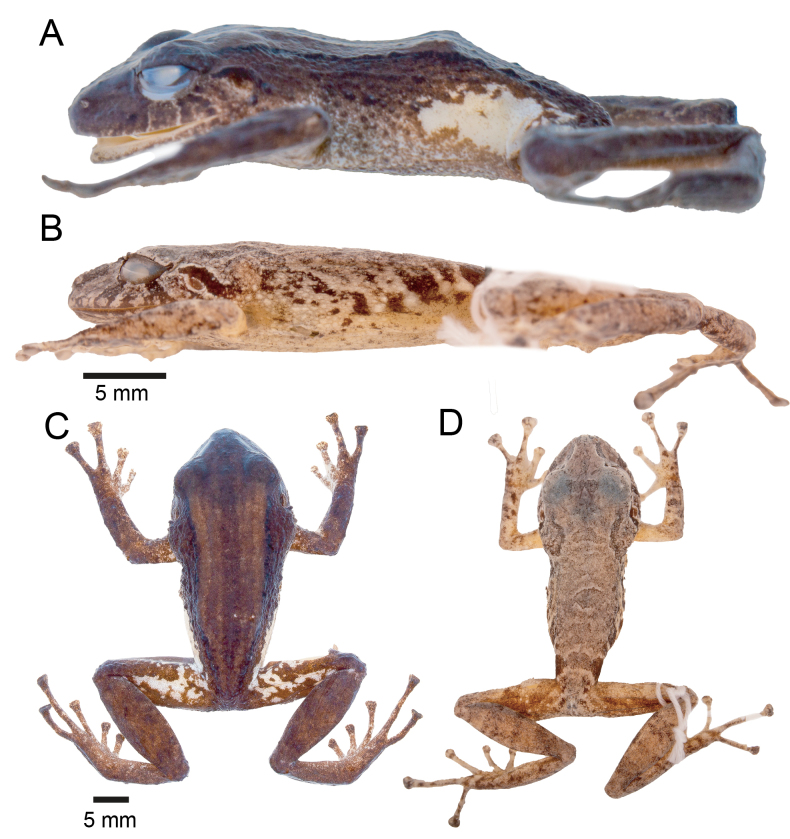
Comparison between the holotype of *Pristimantis etsa* sp. nov. ZSFQ 6188, SVL = 32.5 mm, and the holotype of *P. nangaritza*QCAZ 41710, SVL = 32.4 mm. **A, C**. *Pristimantis etsa* sp. nov.; **B, D**. *P. nangaritza*. Photographs of *P. nangaritza* by Santiago R. Ron; photographs of *P. etsa* sp. nov. by Elías Figueroa-Coronel.

**Figure 9.**
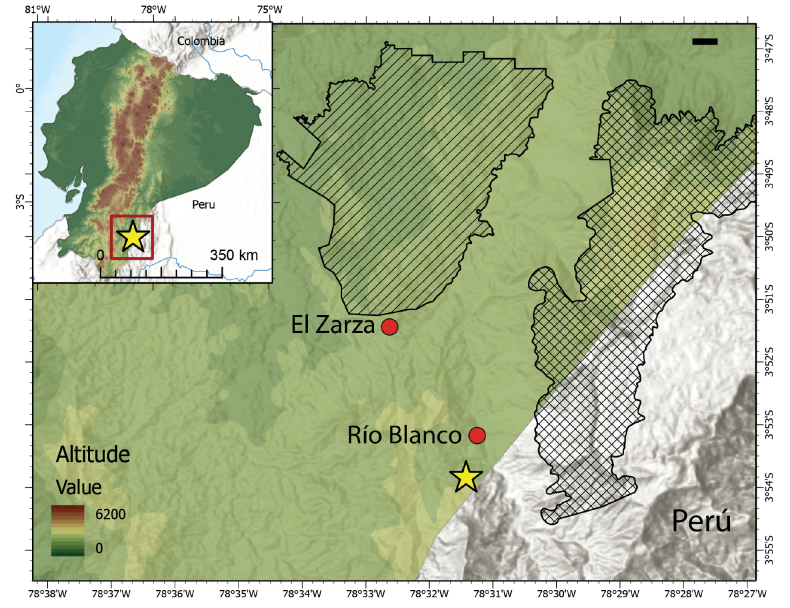
Map of Ecuador showing the type locality of *Pristimantis etsa* sp. nov. (yellow star). Protected areas are indicated with line patterns: Refugio de Vida Silvestre El Zarza with single diagonal lines, and Bosque Protector Cordillera del Cóndor with crossed diagonal lines. Map by Emilia Peñaherrera-Romero.

Figs 3, 4, 5, 6, 7, 8, 9

##### Proposed standard English name.

Etsa Rain Frog.

##### Proposed standard Spanish name.

Cutín de Etsa.

##### Generic placement.

The new species is assigned to *Pristimantis* based on the presence of a differentiated tympanic membrane, S-shaped adductor muscles, and expanded terminal discs on digits bearing well-defined circumferential grooves (Hedges et al. 2008).

##### Type material.

***Holotype*** (Figs 3, 4, 6–8) • ZSFQ 6188, adult female, cerca de la comunidad de Río Blanco, Cordillera del Cóndor (3°54'17"S, 78°30'27"W, 1655 m), parroquia Paquisha, cantón Paquisha, provincia de Zamora Chinchipe, República del Ecuador, collected on 14 August 2023 by Carolina Reyes-Puig, David Brito-Zapata, David Báez, Elías Figueroa-Coronel, and Juan Hurtado. ***Paratype*** (Fig. 5) • ZSFQ 6189, adult male, near the type locality (3°54'59"S, 78°29'31"W, 1830 m), collected on 15 August 2023 by Elías Figueroa-Coronel, Carolina Reyes-Puig, David Brito-Zapata, David Báez, and Juan Hurtado.

##### Definition.

*Pristimantis etsa* sp. nov. is distinguished from all other *Pristimantis* by the following combination of characters: (1) dorsal skin shagreen; dorsolateral folds composed of rows of low subconical tubercles; paravertebral folds thin; flanks with scattered conspicuous subconical tubercles, aggregated anteriorly and some forming discontinuous rows; ventral skin coarsely areolate with upraised warts; discoidal fold present; cloacal region bearing low subconical tubercles and warts; (2) tympanic membrane and annulus distinct; supratympanic fold covering upper 15% of annulus; two prominent conical postrictal tubercles surrounded by smaller subconical tubercles; (3) snout rounded in dorsal and lateral views, with small, low apical papilla; lips slightly flared; (4) upper eyelid with two or three conspicuous subconical tubercles surrounded by smaller subconical tubercles; IOD > EW; interocular region with one subconical tubercle and smaller internasal tubercles; cranial crests absent; (5) dentigerous processes of vomers present, oblique, five or six teeth on each; (6) males with nuptial pads on base of Finger I, vocal slits and vocal sac absent; (7) finger length III>IV>II>I; discs and pads expanded and truncated; discs twice the corresponding digit width; (8) slightly visible lateral fringes, more pronounced on fingers II–III, circumferential grooves present; (9) forearm tubercles conspicuous and subconical, arranged in two distinct rows: one extending along ventrolateral margin of forearm from base of palmar tubercle, and a second extending along externolateral margin of forearm, posteriorly from carpal articulation (Fig. 6); (10) heel tubercle subconical and conspicuous, surrounded by multiple subconical tubercles; two or three subconical outer tarsal tubercles with smaller rounded tubercles; inner tarsal fold extends from inner metatarsal tubercle to ¼ tarsus; (11) inner metatarsal tubercle ovoid and 6–7× the size of outer metatarsal tubercle, which is subconical to ovoid; (12) toe length IV>V>III>II>I; narrow lateral fringes on toes III–V forming a basal membrane; discs and pads similar in size to those of hands; discs III–V more dilated than I–II, equally truncated, distal edge of Toe V reaching distal subarticular tubercle of Toe III; (13) in life, dorsum dark brown with longitudinal stripes lighter brown and mid-dorsal stripe dark brown; subocular stripes, labial and posterior leg bars dark brown; flanks brown with yellow blotch on groin of female with spots of the same coloration extending to the upper and posterior regions of the thighs (pale cream brown suffusion in male); venter cream with small brown splattered spots, denser towards the center of venter; iris copper-golden with thin dark reticulations and broad reddish-brown transverse band (Fig. 7); (14) SVL 32.5 mm in one adult female and 18.3 mm in one adult male. Call unknown.

##### Diagnosis.

The diagnosis is summarized in Table 1, and a visual comparison of *P. etsa* sp. nov. and *P. nangaritza* is shown in Fig. 8. The species included in the comparative diagnosis were selected because they are phylogenetically close to *Pristimantis etsa* sp. nov. within the *P. cryptomelas* group or because they share one or more externally similar characters, especially colored groin, ulnar tuberculation, and occurrence in the Cordillera del Cóndor, southern Ecuador, or adjacent northern Peru. *Pristimantis etsa* sp. nov. is distinguished from these congeners by the following combination of characters: snout rounded in dorsal view and profile; dorsolateral folds conspicuous, composed of subconical tubercles; outer forearm with two distinct rows of tubercles along ventrolateral and externolateral margins; and groin with a yellow blotch and yellow spots in the female and pale cream-brown in the male. The presence of conspicuous dorsolateral folds separates *P. etsa* sp. nov. from *P. verrucosus*, *P. plateado*, *P. aquilonaris* Lehr, Aguilar, Siu-Ting & Jordán, 2007, *P. bellator* Lehr, Aguilar, Siu-Ting & Jordán, 2007, *P. nigrogriseus* (Andersson, 1945), *P. nangaritza*, *P. gagliardoi*, *P. spinosus*, and *P. cryptomelas*, all of which lack dorsolateral folds. It differs from *P. daquilemai* Brito-Zapata, Reyes-Puig, Cisneros-Heredia, Zumel & Ron, 2021 by having conspicuous dorsolateral folds composed of subconical tubercles, a rounded snout, and two distinct rows of forearm tubercles along the ventrolateral and externolateral margins, whereas *P. daquilemai* has thin dorsolateral folds, “› ‹”-shaped scapular folds, and a subacuminate snout. The groin coloration of *P. etsa* sp. nov. further distinguishes it from species with dark or differently pigmented groins, including *P. nigrogriseus*, which has a dark brown to black groin with large yellow spots; *P. aquilonaris*, which has a blackish brown groin with yellowish-orange or reddish-orange flecks; *P. nangaritza*, which has a brown groin with or without minute pale flecks; *P. spinosus*, which has a black groin with white spots; and *P. cryptomelas*, which has a black groin. It also differs from *P. bellator*, *P. gagliardoi*, *P. verrucosus*, and *P. plateado* by the combination of conspicuous dorsolateral folds, two distinct rows of forearm tubercles, and yellow groin blotches in the female, rather than a yellow groin with brown flecks, pink groin, or reddish orange groin coloration in some individuals.

**Table 1.** Morphological comparison of *Pristimantis etsa* sp. nov. with geographically proximate and morphologically similar species.

Species	Female SVL (mm)	Snout, dorsal / profile	Dorsolateral folds	Forearm tubercles	Groin coloration	Distribution	Source
*P. etsa* sp. nov.	32.5	Rounded/rounded	Present, composed of subconical tubercles	Two distinct rows along ventrolateral and externolateral forearm margins	Female with yellow blotch and yellow spots; male with pale cream-brown groin	Cordillera del Cóndor, Zamora Chinchipe, Ecuador	This study
*P. nigrogriseus*	To 30.2	Moderately long, subacuminate/rounded to truncate	Absent	Absent	Dark brown to black with large yellow spots	Eastern slopes of the Cordillera Oriental of the Andes	Andersson 1945; Lynch 1979; Coloma and Duellman 2025
*P. daquilemai*	15.5–19.0	Subacuminate/rounded	Thin dorsolateral folds; “› ‹”-shaped scapular folds	Present, conspicuous, conical	Orange or yellow spots	Cordillera del Cóndor, Zamora Chinchipe, Ecuador	Brito-Zapata et al. 2021
*P. aquilonaris*	19.4–23.0	Long, acuminate/rounded	Absent	Present	Blackish brown with yellowish-orange or reddish-orange flecks	Cordillera de Huancabamba, northern Peru	Lehr et al. 2007
*P. bellator*	17.2–24.3	Long, round/round	Absent	Ulnar tubercles small, low	Yellow with brown flecks	Cordillera de Huancabamba, northern Peru	Lehr et al. 2007
*P. nangaritza*	To 32.4	Moderately long, subacuminate/rounded	Absent	Ulnar tubercles low	Brown, with or without minute pale flecks	Alto Nangaritza, Zamora Chinchipe, Ecuador	Páez and Ron 2019; Coloma and Duellman 2025
*P. gagliardoi*	To 33.6	Rounded/rounded	Absent; suprascapular dermal ridges present	Small	Pink	Cañar and Morona Santiago, Ecuador	Bustamante and Mendelson 2008; Coloma and Duellman 2025
*P. verrucosus*	24.9	Acuminate/rounded	Absent; low middorsal fold present	Present	In some individuals, reddish orange	Reserva Biológica Cerro Plateado, Zamora Chinchipe, Ecuador	Székely et al. 2026b
*P. plateado*	27.2–29.9	Rounded/rounded	Absent	Low	In some individuals, reddish orange	Reserva Biológica Cerro Plateado, Zamora Chinchipe, Ecuador	Székely et al. 2026b
*P. spinosus*	To 34.5	subacuminate/truncate	Absent	Present, subconical	Black with white spots	Azuay, Morona Santiago, and Zamora Chinchipe, Ecuador	Lynch 1979; Coloma and Duellman 2025
*P. cryptomelas*	To 38.6	Subacuminate with pointed tip/rounded	Absent	Ulnar tubercles prominent	Black	Loja, Morona Santiago, and Zamora Chinchipe, Ecuador	Lynch 1979; Coloma and Duellman 2025

##### Description of holotype.

Adult female, 32.5 mm SVL (Figs 3, 4, 6, 7), head wider than long, HW 33% of SVL; EN 10% of SVL, ED 12% of SVL; snout rounded in dorsal and lateral views; nostrils dorsolaterally directed; canthus rostralis concave in dorsal and lateral views; lips slightly flared. Upper eyelid bearing three conspicuous subconical tubercles, surrounded by 12 smaller, rounded to subconical tubercles, denser on the posterior part of the eyelid; a row of low, rounded tubercles present along the outer edge of the eyelid. Interorbital area flat, with a tiny round tubercle (EW 51% of IOD). Cranial crests absent. Tympanic membrane differentiated from surrounding skin; tympanic annulus rounded. Supratympanic fold covering 15% of the upper tympanic annulus (Fig. 4C); two highly conspicuous postrictal tubercles forming a ridge, surrounded by several smaller tubercles. Choanae small and oval, not concealed by palatal shelf; vomerine processes present, semicircular, bearing six teeth. Tongue longer than wide, ~60% attached to the floor of the mouth, posteriorly notched.

Dorsal skin shagreen, with two dorsolateral folds formed by tubercles: an upper fold extending from the postocular region to the cloaca, and a lower fold extending from the posterior scapular region to the inguinal region; two thin parallel paravertebral folds extending from the interorbital region to the ilium, more evident in life (Figs 7, 8). Flanks with scattered conspicuous subconical tubercles, aggregated anteriorly, some arranged in discontinuous rows. Three large subconical tubercles present on each flank, denser in the axillary region; limbs tuberculate in dorsal view; a row of low, rounded tubercles present along the dorsal surface of the forearm; small subconical tubercles present on the dorsal surfaces of hands and feet. Venter coarsely areolate; discoidal fold weakly defined, covering 95% of the abdomen from the level of forelimb insertion to near the level of hind limb insertion. Thoracic fold thin; pericloacal region with multiple subconical tubercles of different sizes.

Palmar tubercle bilobed; thenar tubercle elongated and directed outward; subarticular tubercles prominent and subconical, strongly pronounced on Fingers I and II; two subarticular tubercles present on Fingers III and IV, all rounded. Disc of Finger III reaching the distal subarticular tubercle of Finger IV. Supernumerary tubercles present, most evident at the base of Finger III, all rounded (Fig. 4A). Two distinct rows of forearm tubercles evident and subconical: one extending from the base of the palmar tubercle along the ventrolateral margin of the forearm, and a second extending along the externolateral margin of the forearm.

Hind limbs slender and long (TL 51% of SVL); heel bearing two large subconical tubercles and several smaller subconical tubercles; three small, rounded tubercles present on the inner tarsus; a row of low, rounded tubercles present along the tarsus.

Relative length of toes IV > V > III > II > I; basal webbing present as lateral fringes on Toes III, IV, and V; discs and pads on toes similar in size to those on fingers; discs of Toes III, IV, and V more expanded than those of Toes I and II, all equally truncate. Supernumerary tubercles present at the base of all toes, more aggregated on Toes IV and V (Fig. 4B).

##### Coloration of holotype in preservative.

Dorsum brown with vertebral and dorsolateral dark brown stripes, flanks dark brown; groin with conspicuous white blotches with small brown spots extending towards the dorsal surfaces of thighs. Dorsal surfaces of limbs brown with small cream blotches on hands and feet, mainly on fingers I-III and toes I-IV, venter cream with abundant small dots forming brown blotches, more concentrated on ventral surfaces of limbs. Iris dark gray.

##### Coloration of holotype in life.

(Fig. 7) dorsum brown with darker brown longitudinal stripes extending along the dorsolateral rows of tubercles; axilla and shoulder with inconspicuous yellow spots. In dorsolateral view, head brown with a small yellow patch on the lower half of the tympanum. Labial stripes pale cream with small yellow accents. Groin and flanks with conspicuous large yellow spots, extending from the mid-flanks to nearly the entire surface of the thighs. Limbs dark brown, paler than the dorsum. Iris pale bronze with a reddish-brown horizontal bar and a narrow black median vertical streak and black reticulations. Venter and throat pale creamy-yellow; venter with scattered dark brown spots, whereas the throat bears larger black blotches. Ventral surfaces of hands with small, inconspicuous yellow spots.

##### Measurements (in mm) of holotype.

SVL = 32.5; HW = 13.5; HL = 11.6; ED = 4.0; EW = 3.5; IOD = 6.9; IND = 2.6; EN = 3.3; TL = 16.7; HaL = 9.8; FL = 15.7

##### Variation.

Measurements of the adult male paratype ZSFQ 6189 are as follows, in mm: SVL = 18.3; HW = 7.2; HL = 7.0; ED = 2.8; EW = 2.1; IOD = 4.0; IND = 1.5; EN = 2.1; TL = 10.1; HaL = 6.2; FL = 9.8. The male paratype differs from the female holotype by lacking a conspicuous yellow inguinal blotch; its groin is light brown with dark brown spots and lacks yellow ventral coloration. In life, the male paratype has a darker overall coloration, dark brown lateral bands in the anterior part of the inguinal region, black spots posterior to the eyelids, and a dark brown mid-ventral stripe. The snout of the male paratype appears more angular in lateral view than that of the female holotype, which is rounded. In both specimens, the rostral papilla is less evident in preservative than in life, and tubercles and folds are less conspicuous in preserved specimens.

##### Etymology.

The specific epithet *etsa* is a noun in apposition derived from the Shuar language. Among the Shuar people, an indigenous nationality inhabiting eastern Ecuador and northern Peru, including parts of the Cordillera del Cóndor, Etsa is a powerful anthropomorphic being whose primary manifestation is the sun. In Shuar cosmology, Etsa acts as a cultural transmitter who endows animals and people with essential skills, such as hunting techniques, restores life to forest birds, and upholds moral order (Pellizaro 1984; Barrueco 1985).

##### Distribution and natural history.

*Pristimantis etsa* sp. nov. is known from two nearby localities, near the Río Blanco hamlet, Paquisha parish, Paquisha canton, Zamora Chinchipe province, Ecuador (Fig. 9). The species inhabits Low Montane Evergreen Forest (MAE 2013) from 1655 to 1830 m a.s.l. on the western slopes of the Cordillera del Cóndor. Both specimens of the type series were active at night (20:00–22:00 h), over shrub leaves, 1.2–2 m above ground. The holotype was found in a swampy habitat, syntopic with centrolenids such as *Chimerella mariaelenae* (Cisneros-Heredia & McDiarmid, 2006), and hylids such as *Dendropsophus minutus* (Peters, 1872), and *Callimedusa ecuatoriana* (Cannatella, 1982). The individual was located in a 0.32-km^2^ patch of selectively logged forest surrounded by pastures. The male paratype was discovered 2.2 km away in a straight line to the southeast, within a much larger continuous forest. We did not detect *P. etsa* sp. nov. during surveys conducted in the lowest areas of Río Blanco (at 1560 m) or higher up in regions covered by Evergreen Shrubland and Montane Grassland or Evergreen Montane Forest on Sandstone Plateaus.

##### Conservation status.

The type locality of *Pristimantis etsa* sp. nov. lies in the Río Blanco sector of the Cordillera del Cóndor (Fig. 9), an area undergoing rapid and severe anthropogenic transformation. Legal and illegal mining are the primary drivers of habitat loss and degradation. Illegal operations employ highly destructive placer and underground extraction methods along riverbanks and adjacent mountains, causing extensive soil disturbance and water pollution. In addition, large-scale industrial extraction at the Fruta del Norte gold mine and associated infrastructure has reshaped land-use dynamics across the region, which currently includes 28 active metallic mining concessions and three concessions for construction materials, together covering 64,453 ha; mining activities are projected to continue at least until 2031, with the possibility of a 25-year extension (Hochstein et al. 2023). Agriculture and cattle ranching further contribute to the progressive removal and fragmentation of forest along the foothills and lower slopes, intensifying the isolation and degradation of remaining habitat fragments.

Despite substantial survey effort at the type locality—15 days of fieldwork in June 2025, 10 days in August 2023, and four additional four-day campaigns between 2021 and 2022—only two individuals of *P. etsa* sp. nov. have been detected. Extensive herpetological surveys in nearby areas of the Cordillera del Cóndor have not produced additional records of the species. Using the georeferenced records and the GeoCAT tool (Bachman et al. 2011), and following IUCN mapping standards with a 2 × 2 km grid, we estimated an area of occupancy (AOO) of 8 km^2^, corresponding to two occupied grid cells. The species is therefore known from a single threat-defined location exposed to intense and ongoing mining and agricultural pressures.

*Pristimantis etsa* sp. nov. clearly meets the conditions for listing under criterion D2: it has a very restricted AOO (typically < 20 km^2^) and occurs at a single location where plausible, well-documented threats could rapidly drive the taxon to a higher risk category, or even towards extinction, in a short time frame. On this basis, and despite the absence of quantitative data on population size or long-term demographic trends, we recommend that *P. etsa* sp. nov. be assessed as Vulnerable (VU) under the criterion D2. Further surveys, ecological studies, and monitoring are urgently needed to refine estimates of its distribution, evaluate population dynamics, and determine whether it may warrant uplisting if habitat loss and degradation at Río Blanco continue or intensify.

## Discussion

The discovery of *Pristimantis etsa* sp. nov. adds to the growing assemblage of narrowly distributed terrestrial-breeding frogs known from the Cordillera del Cóndor and further emphasizes the relevance of this mountain range as a center of amphibian diversification in southeastern Ecuador. The new species is currently known only from two nearby localities in the Río Blanco sector, where it inhabits low montane evergreen forest at elevations between 1655 and 1830 m. Its restricted distribution, distinctive phenotype, and phylogenetic placement support its recognition as an independently diagnosable lineage within the subgenus *Huicundomantis* and the *P. cryptomelas* species group. Páez and Ron (2019) defined the *P. cryptomelas* species group as a strongly supported clade characterized by the presence of postocular folds, absence of dorsolateral folds, absence of cranial crests, except for low crests in *P. spinosus*, prominent tympanic membrane and tympanic annulus, prominent eyelid tubercles, dentigerous processes of vomers, conspicuous tubercles on the heel and tarsus, lateral fringes on fingers and toes, broadly expanded digital discs, basal webbing between toes, and distinctive coloration patterns on the groins and concealed surfaces of thighs. The original description of the group included five species: *P. cryptomelas*, *P. gagliardoi*, *P. muscosus*, *P. spinosus*, and *P. nangaritza*. Subsequent taxonomic work has expanded the known diversity of this lineage, including recently described taxa from the Cordillera del Cóndor, such as *P. verrucosus* and *P. plateado* (Székely et al. 2026b). The placement of *P. etsa* sp. nov. within this group is consistent with the expectation of Páez and Ron (2019) that additional collecting and genetic studies, particularly in southern Ecuador and northern Peru, would reveal further species diversity within the clade. Our phylogenetic analysis recovers *P. etsa* sp. nov. as sister to a clade comprising *P. verrucosus*, *P. nangaritza*, and *P. plateado*. This topology is broadly congruent with previous phylogenetic hypotheses for *Huicundomantis* proposed by Páez and Ron (2019) and Székely et al. (2026b), although it differs in the internal arrangement of the *P. cryptomelas* group. In our tree, *P. verrucosus* occupies a basal position within the sister clade of *P. etsa* sp. nov., whereas *P. nangaritza* is recovered as sister to *P. plateado*. This phylogenetic placement is also geographically coherent, because members of the *P. cryptomelas* group occur along the eastern Andean slopes of southern Ecuador and northern Peru, including montane and foothill forests in Cañar, Loja, Morona Santiago, and Zamora Chinchipe provinces (Páez and Ron 2019). The occurrence of *P. etsa* sp. nov. in the Río Blanco sector therefore reinforces the Cordillera del Cóndor as an important area of diversification within *Huicundomantis*, with this species being the first described from the locality.

Although 16S is one of the most widely used mitochondrial markers in amphibian systematics, short fragments may provide limited resolution when interpreted in isolation. Consequently, genetic distances should not be treated as universal thresholds for species delimitation, but rather as one line of evidence within an integrative taxonomic framework that also considers diagnostic morphology, phylogenetic placement, geographic distribution, and direct comparison with closely related taxa (Vences et al. 2005; Chan et al. 2022). In this context, the molecular evidence available for *P. etsa* sp. nov. is concordant with morphology and supports its recognition as a distinct evolutionary lineage.

Morphologically, *P. etsa* sp. nov. agrees with most characters used by Páez and Ron (2019) to define the *P. cryptomelas* group, including the absence of cranial crests, distinct tympanic structures, prominent eyelid tubercles, vomerine teeth, conspicuous heel and tarsal tubercles, lateral fringes, expanded digital discs, basal toe webbing, and contrasting coloration on concealed surfaces. Nevertheless, it differs from the previous definition of the group by having conspicuous dorsolateral folds and lacking postocular folds.

The distinction of *P. etsa* sp. nov. from its closest relatives is supported by a combination of characters rather than by any single trait. The species differs from *P. nangaritza*, *P. verrucosus*, and *P. plateado* mainly in the presence of dorsolateral folds, the arrangement of forearm tubercles and the contrasting yellow inguinal coloration observed in the female holotype. In particular, the yellow blotch in the groin and the yellow marks extending toward the posterior surfaces of the thighs contrast with the more continuous or less conspicuous coloration observed in these taxa. Similar chromatic variation involving concealed body regions has been documented in *Pristimantis* (García-Gómez et al. 2022), but the limited sample size of *P. etsa* sp. nov. prevents us from interpreting this condition as sexual dichromatism. Therefore, groin coloration is treated here as a diagnostic character observed in the available type material, pending additional specimens that allow evaluation of intraspecific, ontogenetic and sex-associated variation.

Beyond its taxonomic relevance, *P. etsa* sp. nov. highlights positional variation in forearm tubercles that may be relevant for the descriptive terminology used in *Pristimantis*. In the new species, two distinct series of tubercular structures occur on the forearm. One row is positioned ventrolaterally and extends from the base of the palmar tubercle, corresponding to the position traditionally described for ulnar tubercles by Lynch and Duellman (1997) and Duellman and Lehr (2009). A second row is located externally on the forearm, separated from the palmar tubercle, and extends along the outer margin of the forearm. In *P. etsa* sp. nov., the row along the external forearm margin forms a low, partially coalesced ridge. Comparable structures occur in other species, although they may appear separated, diffuse, or less conspicuous. The simultaneous occurrence of two externally visible rows of forearm tubercles also occurs in *P. galdi* Jiménez de la Espada, 1870, *P. albujai* Brito-M, Batallas-Revelo & Yánez-Muñoz, 2017, and *P. colonensis* (Mueses-Cisneros, 2007), suggesting that variation in the number and position of forearm tubercle rows may occur across species. This distinction has broader implications for morphological terminology in *Pristimantis*. The traditional term “ulnar tubercles” may have been applied historically to more than one anatomical structure, potentially obscuring differences in position, homology, and phylogenetic informativeness. In species described as having a single row of “ulnar tubercles,” it may be unclear whether the described tubercles occupy the ventrolateral margin, the externolateral margin, or another position on the forearm. A broader comparative assessment across the genus is therefore needed to determine whether some historical references to “ulnar tubercles” require reinterpretation. Future studies should also evaluate intraspecific variation in the number, position, shape, and size of forearm tubercles, since positional variation was observed in other members of the genus, such as *P. galdi* and *P. gualacenio* Urgilés, Sánchez-Nivicela, Nieves & Yánez-Muñoz, 2014.

The discovery of *P. etsa* sp. nov. also contributes to the broader recognition of the Cordillera del Cóndor as a center of lineage diversification and microendemism. This mountain range has a complex geological history, strong altitudinal zonation, and distinctive sandstone-associated habitats that have contributed to high levels of endemism across multiple groups. Recent taxonomic studies have described or documented narrowly distributed lineages of plants (Vélez-Abarca et al. 2021; Moya et al. 2022), invertebrates (Pazmiño-Palomino et al. 2025), mammals (Luis and Patterson 1996; Brito et al. 2021), reptiles (Parra et al. 2020; Brito-Zapata et al. 2023), and amphibians (Ron et al. 2018; Brito-Zapata et al. 2021; Székely et al. 2023). With the description of *P. etsa* sp. nov., fourteen species of *Pristimantis* are now known to be narrowly distributed in the Cordillera del Cóndor: *P. barrigai* Brito & Almendáriz C., 2018, *P. daquilemai*, *P. ledzeppelin* Brito-Zapata & Reyes-Puig, 2021, *P. minimus* Terán-Valdez & Guayasamin, 2010, *P. muranunka* Brito M., Almendariz-C., Batallas R. & Ron, 2017, *P. muscosus*, *P. nangaritza*, *P. nanus* Zumel, Buckley & Ron, 2021, *P. paquishae* Brito-M., Batallas-R. & Velalcázar, 2014, *P. plateado*, *P. pramukae* Zumel, Buckley & Ron, 2021, *P. verrucosus*, *P. yantzaza* Valencia, Dueñas, Székely, Batallas-R. & Pulluquitín, 2017, and *P. etsa* sp. nov. This concentration of geographically restricted species reinforces the interpretation of the Cordillera del Cóndor as a hotspot of fine-scale diversification in the genus *Pristimantis*.

The Río Blanco hamlet of the Cordillera del Cóndor is undergoing rapid anthropogenic transformation driven primarily by legal and illegal mining, agriculture, and cattle ranching. Illegal mining operations employ highly destructive extraction methods along riverbanks and adjacent slopes, producing soil disturbance, forest degradation, and water pollution. At the same time, large-scale industrial extraction, including the Fruta del Norte gold mine and associated infrastructure, has reshaped land-use dynamics across the region (Chicaiza and Yánez 2013; Roy et al. 2018). Agriculture and cattle ranching further contribute to the progressive removal and fragmentation of forest along foothills and lower montane slopes, intensifying the isolation of remaining habitat fragments. Conservation of *P. etsa* sp. nov. will depend on maintaining the ecological integrity of low montane evergreen forests in Río Blanco and adjacent areas. More broadly, the species illustrates the need to incorporate fine-scale amphibian endemism into environmental impact assessments, land-use planning, and mining policy in the Cordillera del Cóndor. Conservation strategies in this region should therefore move beyond generalized habitat protection and explicitly consider the narrow distributions, ecological specificity, and taxonomic singularity of terrestrial-breeding frogs inhabiting these montane landscapes.

## Supplementary Material

XML Treatment for Pristimantis etsa

## Appendix 1

**Examined specimens**. *Pristimantis colonensis* (1). ECUADOR: Sucumbíos Province, Santa Bárbara (ZSFQ 2144). *Pristimantis croceoinguinis* (9). ECUADOR: Pastaza Province, Curaray Medio (ZSFQ 262); Reserva Río Anzu (ZSFQ 1329). Orellana Province, Tiputini Biodiversity Station (ZSFQ 3127, 3128, 6781); Pompeya (ZSFQ 3129–3131). *Pristimantis daquilemai* (26). ECUADOR: Zamora Chinchipe Province, Río Blanco (ZSFQ 1201–1212, 1876, 1883, 1884, 6193–6196, 7473–7477, 7479, 7480). *Pristimantis etsa* sp. nov. (2). ECUADOR: Zamora Chinchipe Province, Río Blanco (ZSFQ 6188, 6189). *Pristimantis gagliardoi* (104). ECUADOR: Cañar Province, Reserva Mazar, La Libertad; La Libertad WGS 17M; Mazar, Reserva Mazar, campamento La Libertad; Reserva Mazar, campamento La Libertad; Reserva Mazar, Rivera, Parque Nacional Sangay, nuevo sendero desde el campamento de La Libertad hasta el valle de Gasualpampa; Reserva Ecológica Mazar, Comuna La Libertad (QCAZA 27102–27104, 27434, 27436, 27568–27571, 27577–27583, 29557–29558, 29560–29561, 32623, 32625–32626, 46738, 68173–68251). Morona Santiago Province, Zuñiag, Galgalán, guardianía del Ministerio del Ambiente del Parque Nacional Sangay, 1 km from Laguna Negra, Atillo, toward Macas, Río Tinguichaca (QCAZA 42575). *Pristimantis galdi* (25). ECUADOR: Provincia de Napo, Parque Nacional Sumaco (ZSFQ 790, 811). Zamora Chinchipe Province, Río Blanco (ZSFQ 1185, 6179–6183, 7450, 7451); Yacuambi (ZSFQ 5854); Reserva Biológica Cerro Plateado, Guardianía Romerillo (ZSFQ 7256, 7257, 7267, 7349); Reserva Biológica Cerro Plateado, Rancho Las Tarántulas (ZSFQ 7291, 7370, 7389); Reserva Biológica Cerro Plateado, Rancho Las Tarántulas, Helipuerto (ZSFQ 7298). Tungurahua Province, Reserva Río Zuñag (ZSFQ 1334). Azuay Province, Guarumales (ZSFQ 5855, 5857). Morona Santiago Province, Parque Nacional Río Negro-Sopladora (ZSFQ 5856, 5858, 5859). *Pristimantis gualacenio* (4). ECUADOR: Morona Santiago Province, Parque Nacional Río Negro-Sopladora (ZSFQ 5871–5874). *Pristimantis nangaritza* (18). ECUADOR: Zamora Chinchipe Province, Zurmi, Las Orquídeas, Tepuy, campamento dos, bosque de Tepuy (QCAZA 41704–41710, 41725–41726, 41728–41736). *Pristimantis nigrogriseus* (1). ECUADOR: Tungurahua Province, Reserva Azuay (ZSFQ 4363). *Pristimantis plateado* (7). ECUADOR: Zamora Chinchipe Province, Reserva Biológica Cerro Plateado, Tepuy El Plateado (ZSFQ 7269, 7316, 7342, 7350, 7364, 7378, 7384). *Pristimantis variabilis* (4). ECUADOR: Orellana Province, Tiputini Biodiversity Station (ZSFQ 3124); Parque Nacional Yasuní, km 28 de la carretera Pompeya Sur-Iro (ZSFQ 3126); Carretera Pompeya Sur, Parque Nacional Yasuní (ZSFQ 3132). Provincia de Sucumbíos, Comunidad Nueva Juventud, transectos hacia la Reserva de Producción Faunística Cuyabeno (ZSFQ 3125). *Pristimantis verrucosus* (1). ECUADOR: Zamora Chinchipe Province, Reserva Biológica Cerro Plateado, Tepuy El Plateado (ZSFQ 7272).

## Additional information

### Artificial Intelligence (AI) use

The authors accept full responsibility for the content of the manuscript, including the disclosure of any use of AI.

No AI tools were used in the preparation of this manuscript.

### Author ORCIDs

E. Figueroa-Coronel https://orcid.org/0009-0007-1617-3570

D.F. Cisneros-Heredia https://orcid.org/0000-0002-6132-2738

D. Brito-Zapata https://orcid.org/0000-0003-2100-0555

J.C. Carrión-Olmedo https://orcid.org/0000-0002-4536-6052

C. Reyes-Puig https://orcid.org/0000-0001-7828-8698

### Data availability

All of the data that support the findings of this study are available in the main text.
